# Entropy, Information, and Symmetry; Ordered Is Symmetrical, II: System of Spins in the Magnetic Field

**DOI:** 10.3390/e22020235

**Published:** 2020-02-19

**Authors:** Edward Bormashenko

**Affiliations:** Department of Chemical Engineering, Ariel University, Ariel 407000, Israel; edward@ariel.ac.il; Tel.: +972 074 729 68 63

**Keywords:** entropy, symmetry, ordering, elementary magnets, magnetic field, *j*-fold symmetry

## Abstract

The second part of this paper develops an approach suggested in *Entropy* **2020**, *22*(1), 11; which relates ordering in physical systems to symmetrizing. Entropy is frequently interpreted as a quantitative measure of “chaos” or “disorder”. However, the notions of “chaos” and “disorder” are vague and subjective, to a great extent. This leads to numerous misinterpretations of entropy. We propose that the disorder is viewed as an absence of symmetry and identify “ordering” with symmetrizing of a physical system; in other words, introducing the elements of symmetry into an initially disordered physical system. We explore the initially disordered system of elementary magnets exerted to the external magnetic field $\vec{H}$. Imposing symmetry restrictions diminishes the entropy of the system and decreases its temperature. The general case of the system of elementary magnets demonstrating *j*-fold symmetry is studied. The $T_{j}=\frac{T}{j}$ interrelation takes place, where *T* and $T_{j}$ are the temperatures of non-symmetrized and *j*-fold-symmetrized systems of the magnets, correspondingly.

## 1. Introduction

Entropy is a key concept in the characterization of ordering in physics [1,2], chemistry [3], biology [4,5], and engineering [6]. However, it remains one of the most abstract and least intellectually transparent quantities in physics [7,8,9]. The widespread illustrative interpretation of entropy is “the measure of disorder” in macroscopic systems built from a large number of particles [10]. However, researchers recently criticized the equating of entropy with disorder [8]. In the first part of our manuscript, we suggested that that “ordering” may be related to symmetry, inherent for the physical system [11]. In turn, “chaos” or “disorder” are understood as an absence of symmetry [11]. We have already illustrated this suggestion with the simplest binary 1D and 2D systems built using elementary magnets, which can point only up or down, fixed in a space, and aligned [11]. They form a binary, non-interacting system. We demonstrated that introducing elements of symmetry diminishes the entropy, which is true for 1D and 2D systems built using elementary magnets [11]. In the present work, we generalize the approach reported in [11] for the initially disordered systems of elementary magnets, embedded into the magnetic field$\;\vec{H}$, and symmetrized by the *j*-fold symmetrizing procedure. 

## 2. Symmetry and Entropy of Binary Magnetic Systems Embedded into a Magnetic Field 

### 2.1. Symmetrizing and Entropy of 1D Systems Exposed to Magnetic Field $\vec{H}$

**Figure 1:**
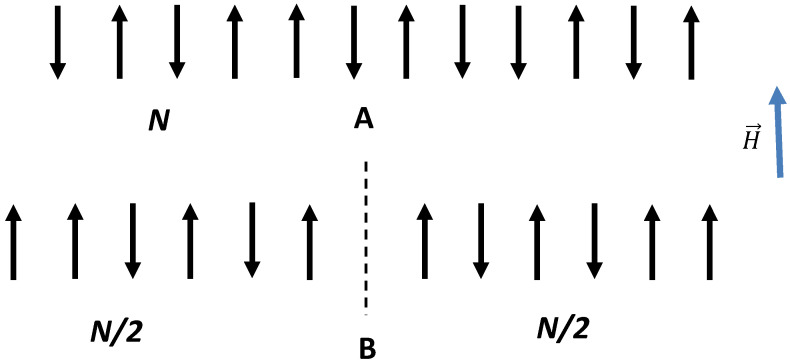
(**A**) The binary 1D system of *N* non-interacting elementary magnets is shown, exposed to external magnetic field $\vec{H}\neq 0$. The spin excess of the system is given by $2m=\frac{1}{2}N+m-\left(\frac{1}{2}N-m\right).$ (**B**) The axis of symmetry shown with a dashed line “arranges” elementary magnets and restricts the number of available configurations of magnets.

First, consider a binary 1D system built using non-interacting magnets (spins) $\vec{\mu }$, illustrated in Figure 1A. We assume that there are *N* separate and distinct sites fixed in a space and aligned, as shown in Figure 1A [11]. Attached to each site is an elementary magnet $\vec{\mu }$, which can point only up or down. The system using magnets is embedded into magnetic field $\vec{H}\neq 0$, leading to spin orientation. The potential energy of a single elementary magnet in the magnetic field is given by:

(1) $$U_{1}=-\vec{\mu }\cdot \vec{H}$$

The magnetic field directs the orientation of the magnets. The configuration of magnets demonstrating spin excess *2m* is defined by Equation (2) (the numbers *N* and *m* are supposed to be even):

(2) $$\frac{1}{2}N+m-\left(\frac{1}{2}N-m\right)=2m$$

corresponding to the configuration where $\frac{1}{2}N+m$ of magnets are oriented “up” and $\frac{1}{2}N-m$ are oriented “down”. The total potential energy of the system of magnets characterized by spin excess *2m* is given by [12,13,14]:

(3) U(2m)=−2mμH

The entropy *S* of this system is given by [12,13,14]:

(4a) $$S\left(N,m\right)=k_{B}lng\left(N,m\right)$$

(4b) g(N,m)≅2N(2πN)12exp(−2m2N)

(4c) $$S\left(N,\;m\right)≅k_{B}\left[Nln2-\frac{1}{2}ln\frac{2}{\pi N}-\frac{2m^{2}}{N}\right]=S_{0}\left(N\right)-\frac{k_{B}U^{2}}{2N\mu^{2}H^{2}}$$

where g(N,m) is the multiplicity function, i.e., the number of states having the same value of *m* [12,13,14]; $S_{0}\left(N\right)=k_{B}\left[Nln2-\frac{1}{2}ln\frac{2}{\pi N}\right].$ Eqs. 4b-c hold for $m\gg 1;N\gg 1;\frac{\left|m\right|}{N}\ll 1$. 

Now, let us restrict the possible configurations of elementary magnets by introducing the symmetry axis, shown with the dashed line in Figure 1B, keeping the spin excess of the system 2*m* and correspondingly its energy *U* the same. After introducing the symmetry axis, only the symmetric configurations of the elementary magnets are available, as depicted in Figure 1B; this implies a decrease in the number of “states” available for the symmetrized system to $g\left(\frac{N}{2},m\right)$. The multiplicity function for the symmetrized, ordered, binary, non-interacting system is given by [12,13,14]:

(5) g(N2,m)≅2N2(2π(N2))12exp(−4m2N)

Hence, the entropy of the symmetrized, ordered, binary, non-interacting system is given by:

(6) $$S_{2}\left(N,m\right)=k_{B}\ln g\left(\frac{N}{2},m\right)≅S_{02}\left(N\right)-\frac{k_{B}U^{2}}{N\mu^{2}H^{2}}$$

where subscript “2” indicates the presence of the axis of symmetry of the second order, and $S_{02}\left(N\right)=k_{B}\left[\frac{N}{2}ln2-\frac{1}{2}ln\frac{2}{\pi \frac{N}{2}}\right]$ takes place. On combining Equations (3)–(6) and with trivial transformations, the following is obtained:

(7) $$S-S_{2}≅k_{B}\left[\frac{N}{2}ln2+\frac{2m^{2}}{N}\right]=k_{B}\left[\frac{N}{2}ln2+\frac{U^{2}}{2N\mu^{2}H^{2}}\right]>0$$

It is to be noted that introducing symmetry decreases the entropy, irrespective of the values of spin excess *2m*, energy of the system *U,* and value of magnetic field $\vec{H}\;$(recall that Equation (7) holds for $m\gg 1;N\gg 1;\frac{\left|m\right|}{N}\ll 1$). The larger the spin excess 2*m,* the stronger a decrease in entropy emerging from symmetrizing. Thus, the generalization of the results reported in [11] is achieved. 

On considering the temperatures of the original *T* and symmetrized *T_2_* systems of magnets, Equations. (4c) and 6 yield [12,13,14]:

(8a) $$\frac{1}{T}={\left(\frac{\partial S}{\partial U}\right)}_{N}=-\frac{k_{B}U}{N\mu^{2}H^{2}}$$

(8b) $$\frac{1}{T_{2}}={\left(\frac{\partial S_{2}}{\partial U}\right)}_{N}=-\frac{2k_{B}U}{N\mu^{2}H^{2}}$$

Recall that U<0 takes place. Interrelation $T_{2}=\frac{1}{2}T\;$takes place; in other words, the symmetrized system of magnets is “colder” than the non- symmetrized one when spin excess and energy of the systems are the same. This result is intuitively expectable. 

### 2.2. Symmetrizing and Entropy of 2D Systems Possessing Axes of Symmetry of Various Orders (j-Fold Symmetry) 

**Figure 2:**
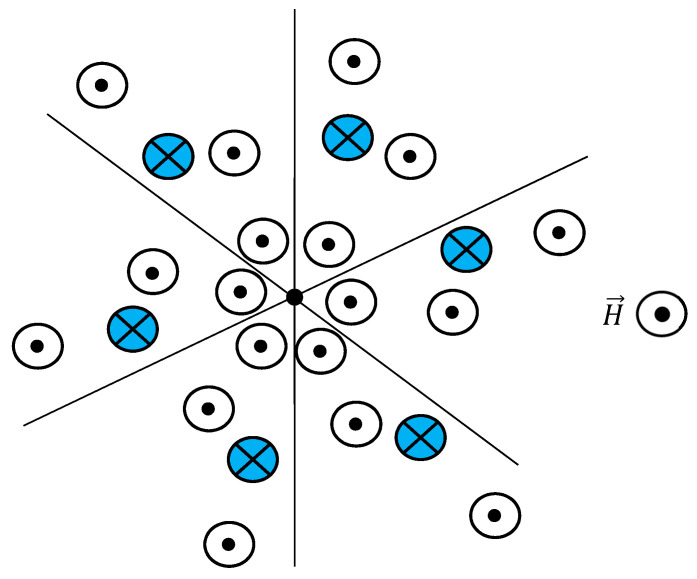
Schematic representation of a system of elementary magnets possessing axis of symmetry to the order of six, embedded into magnetic field $\vec{H}$. Magnetic moments and magnetic field $\vec{H}\;$ are normal to the image plane. Maintaining 6-fold symmetry requires simultaneous re-orientation of six magnets (for example, re-orientation of the magnets, marked in Figure 2 with blue color).

Consider the 2D system of elementary magnets possessing axes of symmetry of the *j*-th order (Figure 2 depicts the sample system of spins with j=6). Again, the number of available states for the *j*-fold-symmetrical system is given by$\;g\left(\frac{N}{j},m\right)$. Indeed, keeping the *j*-fold symmetry requires simultaneous re-orientation of the *j* magnets. The entropy of such a *j*-fold system of magnets is supplied, in turn, by:

(9a) Sj=kBlng(Nj,m)≅kBln{2Nj(2jπN)12exp(−2jm2N)}=S0j(N,j)−2kBjm2N=S0j(N,j)−kBjU22Nμ2H2

(9b) $$S_{0j}\left(N,j\right)=k_{B}\left[\frac{N}{j}ln2+\frac{1}{2}ln\left(\frac{2j}{\pi N}\right)\right]$$

The initial entropy of the 2D non-symmetrical binary system of magnets is given in Equation (4) (2D location of the elementary magnets does not matter; the spin excess of the system 2*m* and its energy *U* are fixed). Combining Equations (9) and (4) yields:

(10) $$S-S_{j}≅k_{B}\left(j-1\right)\left[\frac{N}{j}ln2+\frac{2m^{2}}{N}\right]=k_{B}\left(j-1\right)\left[\frac{N}{J}ln2+\frac{U^{2}}{2N\mu^{2}H^{2}}\right]>0$$

Again, introducing symmetry decreases the entropy, irrespective of the order of the symmetry axis *j*, spin excess *2m*, energy of the system *U,* and value of the magnetic field $\vec{H}\;$(recall that Equations (3), (9a)–(10) hold for $m\gg 1;N\gg 1;\frac{\left|m\right|}{N}\ll 1$). It is easily seen that:

(11) $$\frac{\partial S}{\partial j}=-k_{B}\left(\frac{Nln2}{j^{2}}-\frac{1}{2j}+\frac{2m^{2}}{N}\right)≅-k_{B}\left(\frac{Nln2}{j}+\frac{2m^{2}}{N}\right)<0$$

Equation (11) holds when the condition $\frac{N}{j}\gg 1$ takes place—this means that increase to the order of symmetry axis *j* decreases the entropy of the system. It is also seen from Equation (9a) that Equation (12) is true:

(12) $$\frac{1}{T_{j}}={\left(\frac{\partial S_{j}}{\partial U}\right)}_{N,j}=-\frac{k_{B}jU}{N\mu^{2}H^{2}}\;$$

where $T_{j}$ is the temperature of the system of magnets, possessing axis of symmetry to the order of *j*, i.e., *j*-fold symmetry. Comparing Equations (12) and (8a) results in:

(13) $$T_{j}=\frac{T}{j}$$

Further symmetrizing of the system of magnets “cools” it; moreover, the larger the value of *j,* the cooler the system is. The presented results support the idea that ordering (understood as symmetrizing) decreases the multiplicity of the system and consequently decreases the entropy. 

The obtained results are valid when the condition $\frac{\mu H}{k_{B}T}\ll 1$ holds, as discussed in detail in [14]. The exact expressions should be derived by analysis of the partition function of the system of elementary magnets embedded into the magnetic field. However, the reported considerations qualitatively illustrate the suggested idea: the ordering (“arranging”) may be related to the symmetrizing of a physical system, decreasing its entropy. We already mentioned in the first part of the paper that it is possible that there are other pathways of ordering (“arrangement”) of physical systems, in addition to imposing elements of symmetry; these alternative pathways call for additional physical insights.

## 3. Conclusions

We conclude that the introduction of elements of symmetry orders (arranges) the system of elementary magnets exposed to the external magnetic field and consequently diminishes its multiplicity, entropy, and temperature. The idea is illustrated with a binary system built from elementary non-interacting magnets $\vec{\mu }\;$embedded into magnetic field $\vec{H}$. Symmetrizing of the initially disordered system of *N* magnets diminishes the multiplicity function g(N,m), where 2*m* is the spin excess, and consequently decreases the entropy S(N, m). The simplest 1D exemplification of the binary systems is studied. Introducing two-fold symmetry decreases the entropy, irrespective of spin excess *2m*, energy of the system *U,* and value of the magnetic field $\vec{H}$. The paper also addresses the system of elementary magnets demonstrating *j*-fold symmetry and exposed to magnetic field $\vec{H}$. Symmetrizing decreases the multiplicity and entropy of the system, irrespective of the value of *j*; the condition $\frac{\partial S\left(j\right)}{\partial j}<0\;$was found to be true. The $T_{j}=\frac{T}{j}$ interrelation takes place, where *T* and $T_{j}$ are the temperatures of non-symmetrized and *j*-fold-symmetrized systems of the magnets, correspondingly. Thus, symmetrizing necessarily “cools” the system.
